# The effects of competency-based training model in the training of new nurses: A meta-analysis and systematic review

**DOI:** 10.1371/journal.pone.0277484

**Published:** 2022-11-28

**Authors:** Suqing Chen, Chenxia Zhang, Wanling Li

**Affiliations:** 1 Shanxi Bethune Hospital, Shanxi Academy of Medical Sciences, Tongji Shanxi Hospital, Third Hospital of Shanxi Medical University, Taiyuan, China; 2 Tongji Hospital, Tongji Medical College, Huazhong University of Science and Technology, Wuhan, China; Universidad de Chile, CHILE

## Abstract

**Background:**

It is necessary to evaluate the effects of competency-based training (CBT) model in the clinical training of new nurses, to provide insights to the management of nurses.

**Methods:**

We searched PubMed, EMBASE, Cochrane Library, Web of Science, China National Knowledge Infrastructure (CNKI), China Science and Technology Journal Database, Wanfang and Weipu Databases for the randomized controlled trials (RCTs) on the application effect of CBT in new nurse training up to December 15, 2021. Two investigators independently screened the literature, extracted the data and evaluated the quality of the literatures. RevMan 5.3 software was used for meta-analysis.

**Results:**

A total of 7 RCTs involving 639 new nurses were included. Meta-analyses indicated that CBT improved the ability of clinical nursing care [SMD = 1.52, 95%CI (0.13~2.90), P = 0.03], critical thinking and innovation[SMD = 0.69, 95%CI (0.43~0.95), P<0.001], interpersonal communication[SMD = 0.74, 95%CI (0.51~0.97), P<0.001], professional construction and development[SMD = 1.92, 95%CI (1.41~2.42), P<0.001], level of comprehensive knowledge[SMD = 1.20, 95%CI (0.63~1.76), P<0.001] and level of good personal traits[SMD = 1.89, 95%CI (1.27~2.50), P<0.001].The results of Egger regression tests indicated that there were no statistical biases amongst the synthesized outcomes (all P>0.05).

**Conclusions:**

CBT is beneficial for improving the competency of newly recruited nurses. More RCTs from different population and regions are needed to further elucidate the role of CBT in nurse management.

## Introduction

Newly recruited nurses are most nurses who came to the hospital to participate in clinical nursing work within one year after graduating from a nursing school. They are the new force in the nursing team, and their job competencies not only have an important impact on their future careers, but also are related to the overall sustainable and healthy development of the nursing career [1,2]. However, due to the difference between the school and the hospital environment, when a new nurse changes from a student role to a nurse role, she/he is affected by knowledge, responsibilities, roles and relationships, and may be confused in knowledge and skills, social culture and development [3]. It has been reported that the core and job competence in new nurses are affected by the feelings and experiences of doubt, confusion, and unclear positioning [4]. Studies have reported that effective pre-job training for newly recruited nurses can effectively improve their role transformation and job competence. Therefore, what kind of effective training quality evaluation management methods should be selected to ensure the standardized pre-job training quality of newly recruited nurses and improve the competence of newly recruited nurses, and help newly recruited nurses quickly adapt to the change of roles and become practical and high-quality nursing staff are the important topics for clinical nurse managers [5].

The traditional effect evaluation method mostly adopts the final evaluation model of "one test determines all" [6]. This evaluation model seems simple and convenient, but it is insufficient for the enthusiasm of the trainees to learn and the fairness of the evaluation. In the pre-job training process, in addition to mastering professional knowledge, it also needs to develop its own comprehensive ability and job competence in an all-round way [7]. The comprehensive ability of nurses cannot be judged through the traditional single summative evaluation method, and a complete and objective evaluation system needs to be established [8]. The training model of competency-based training (CBT) is based on the key competency characteristics of the job. Under the guidance of job competency, scientific and reasonable training is carried out, and an effective operating mechanism is used as a guarantee to finally achieve the goal of improving personnel competence [9].

Several previous studies [10,11] have applied the CBT training model to the training and teaching of imaging and sonographers, and achieved good results. In recent years, with the in-depth research on the competency of new nurses, more and more studies on the CBT training model have been reported. The constructed CBT training model can be better applied to the standardized training of hospital nurses because the nurses’ knowledge, skills, comprehensive ability, job competency and training satisfaction have significantly improved [12–14]. However, most studies have reported small sample size, and the results of the studies are still divergent and lack sufficient persuasiveness. Therefore, we aimed to systematically evaluate the effect of the CBT model in the training of new nurses through the method of meta-analysis, to provide evidence-based basis for the clinical training of new nurses.

## Methods

We conducted and reported this systematic review and meta-analysis in compliance with the Statement of Preferred Reporting Items for Systematic Reviews and Meta-Analyses (PRISMA) [15].

### Search strategies

The two authors searched PubMed, EMBASE, Cochrane Library, Web of Science, China National Knowledge Infrastructure (CNKI), China Science and Technology Journal Database, Wanfang and Weipu Databases for published randomized controlled trials (RCTs) on the application effect of CBT in new nurse training. The search terms used in this meta-analysis were: ("competence" OR "competency" OR "training" OR "model" OR "education") AND ("new nurse" OR "new graduate nurses" OR "nurse"). The database retrieval time was from the establishment of the database to December 15, 2021. In the search process, the language was limited to Chinese and English. The reference lists of included studies were searched manually to avoid missing documents.

### Literature inclusion and exclusion criteria

The RCT inclusion criteria for this meta-analysis were as follows: (1) Research type: RCTs regarding the application of the training model based on CBT in the training of new nurses, the language was limited to Chinese and English. (2) Research population: new nurses who had nurse practitioner qualification certificate and had been engaged in nursing work within one year. (3) Intervention measures: the experimental group adopted the training mode based on CBT. The control group was trained using traditional teaching methods. (4) Related outcome indicators were reported.

The literature exclusion criteria for this meta-analysis were as follows: (1) The duplicate publications with the same study data, setting or population; (2) Literature whose original data was not obtained through contacting the corresponding authors with email or telephone.

### Literature screening and data extraction

Two researchers independently searched the above-mentioned database, and independently screened the literature according to the inclusion and exclusion criteria. The agreement rate between the two investigators was 90%. Then they extracted literature information, including basic information (title, author, year of publication, baseline situation), sample size, intervention measures, outcome indicators, etc. In the process of literature inclusion, if there were differences of opinion, it was resolved by the third researcher for arbitration.

### Literature quality evaluation

Two researchers conducted independent quality evaluation of the included RCTs in accordance with the quality standards of the Cochrane Handbook. The evaluation items included: random sequence generation, allocation hiding, whether to blind the researchers and study population, the completeness of the result data, and the selectivity of report research results and other sources of bias. The risk of bias for every item was rated as being low, unclear and high accordingly. After the independent evaluation was completed, the two researchers discussed the results of the evaluation to reach a consensus. If there was disagreement, the third researcher would be consulted to resolve the disagreement.

### Statistical analysis

We used RevMan 5.3 statistical software to conduct statistical analysis. We initially evaluated whether there was clinical heterogeneity in the study with Chi-square(I^2^) test to quantify the amount of dispersion [16]. For homogeneity studies (I^2^ <50%, P>0.1), fixed effects model was selected for analysis; for heterogeneity studies (I^2^ ≥ 50%, P <0.1), random effects model was selected. Outcomes were analyzed and reported as standardized mean differences (SMD) with 95% confidence interval (95%CI). Publication bias were evaluated by using funnel plots, and asymmetry was assessed by performed Egger regression test. For funnel plot asymmetry, P <0.1 was considered as significantly different. In this meta-analysis, P <0.05 indicated that the difference between groups was statistically significant.

## Results

### Literature search results

**Fig 1:**
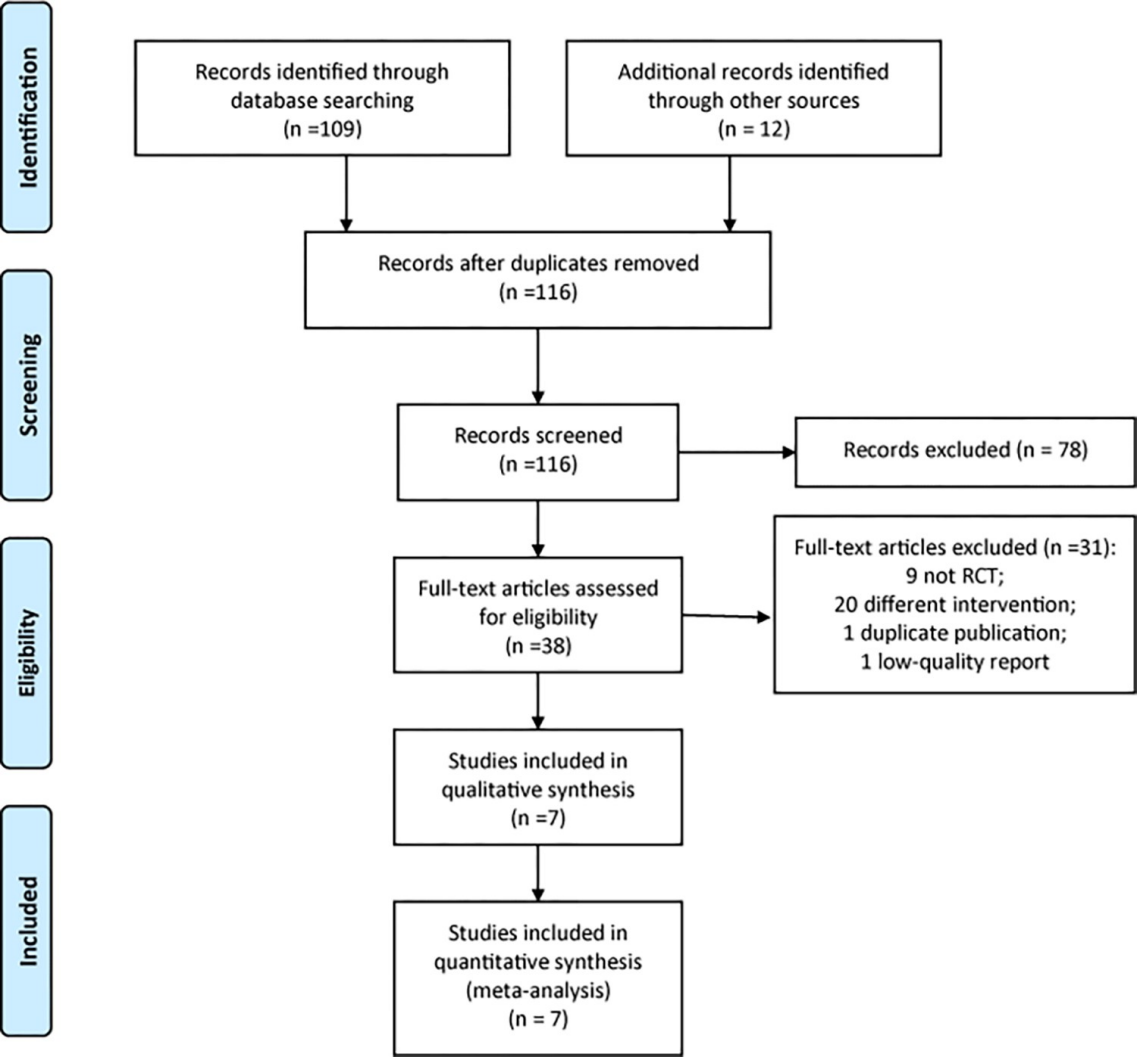
The flow chart of study selection.

A total of 121 documents were obtained through preliminary search, including 80 in English and 41 in Chinese. 5 duplicate documents were removed by Endnote software, 21 English documents and 17 Chinese reports were obtained after reading the title and abstract of the documents. After reading the full text, there were 7 reports that met the inclusion criteria (Fig 1). Therefore, this meta-analysis included a total of 7 RCTs [17–23] for data analysis.

### The basic characteristics and quality evaluation of the included studies

**Fig 2:**
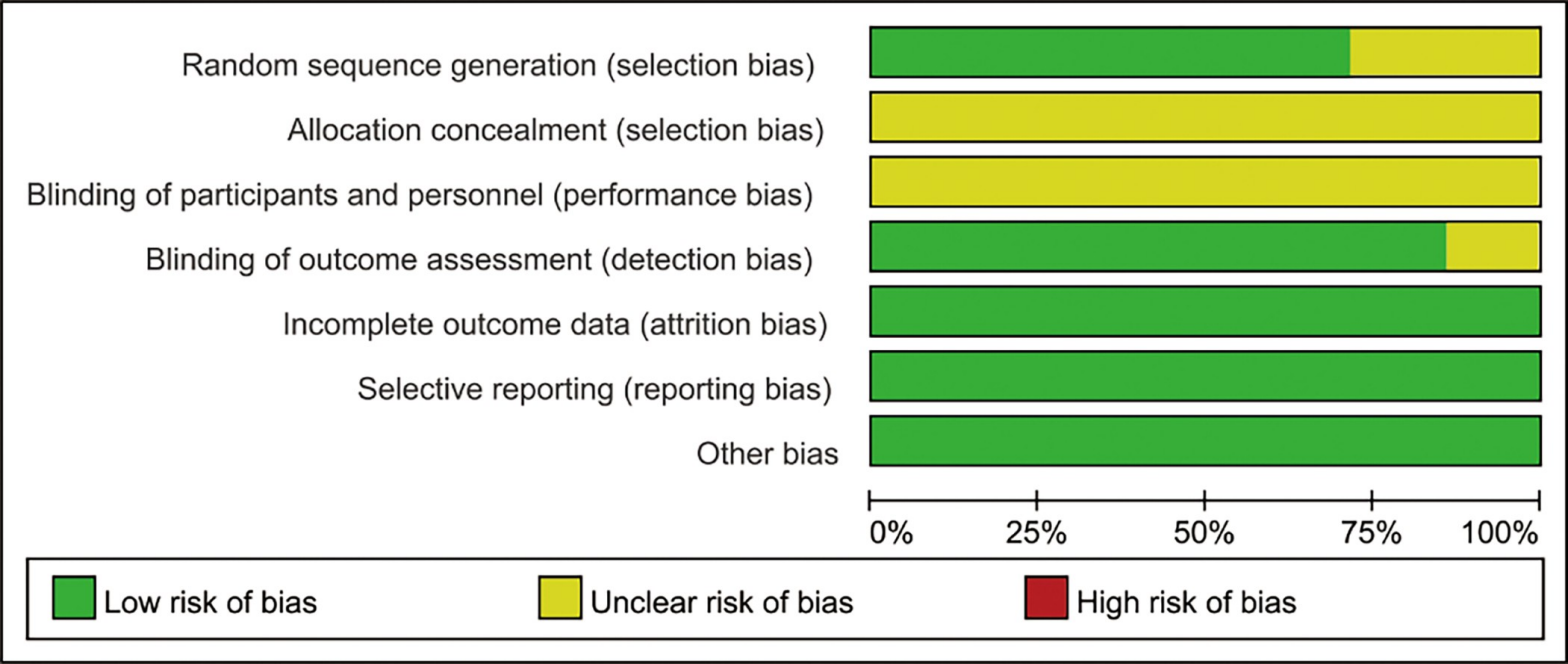
Risk of bias graph.

**Fig 3:**
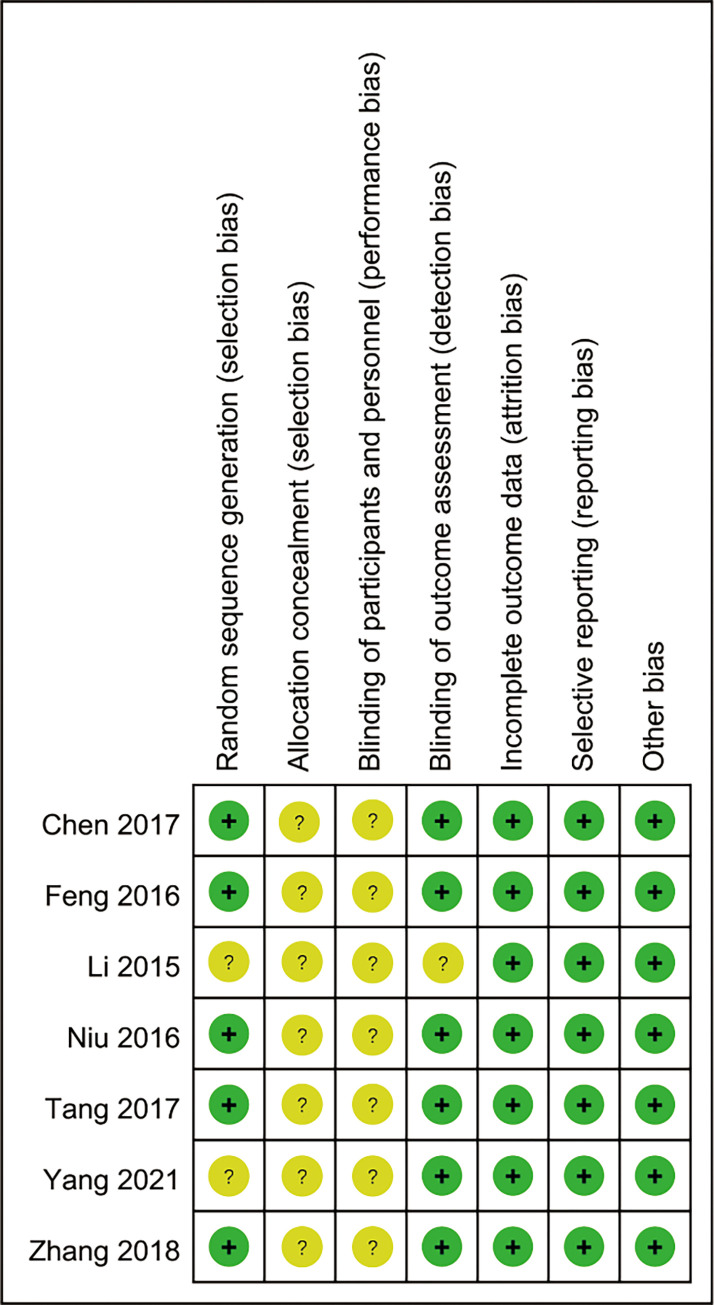
Risk of bias summary.

The basic characteristics of the included RCTs are shown in Table 1. Of the included 7 RCTs [17–23], a total of 639 new nurses were included, involving 320 nurses underwent CBT and 319 underwent traditional training. We strictly evaluate all the included literature in accordance with the Cochrane System Review Manual quality standard. The methodological quality evaluation results of the included studies are shown in Figs 2 and 3.

**Table 1:** The characteristics of included RCTs.

RCT ID	Country	Study population	Sample size		Duration of training(months)	Outcome indicators
			CBT	Control		
Chen 2017	China	New nurses	58	62	12	Level of comprehensive knowledge; level of good personal traits; ability of interpersonal communication; ability of clinical nursing care; ability of professional construction and development; ability of critical thinking and innovation.
Feng 2016	China	New nurses	18	18	12	Ability of interpersonal communication; ability of critical thinking and innovation.
Li 2015	China	New nurses	85	89	6	Level of comprehensive knowledge.
Niu 2016	China	New nurses	36	27	24	Level of comprehensive knowledge.
Tang 2017	China	New nurses in the department of neurology	37	37	7	Ability of interpersonal communication.
Yang 2021	China	New nurses	42	42	6	Level of comprehensive knowledge; satisfaction rate.
Zhang 2018	China	New nurses	44	44	24	Level of good personal traits; ability of interpersonal communication; ability of clinical nursing care; ability of professional construction and development; ability of critical thinking and innovation.

### Meta-analysis

#### Ability of clinical nursing care

**Fig 4:**
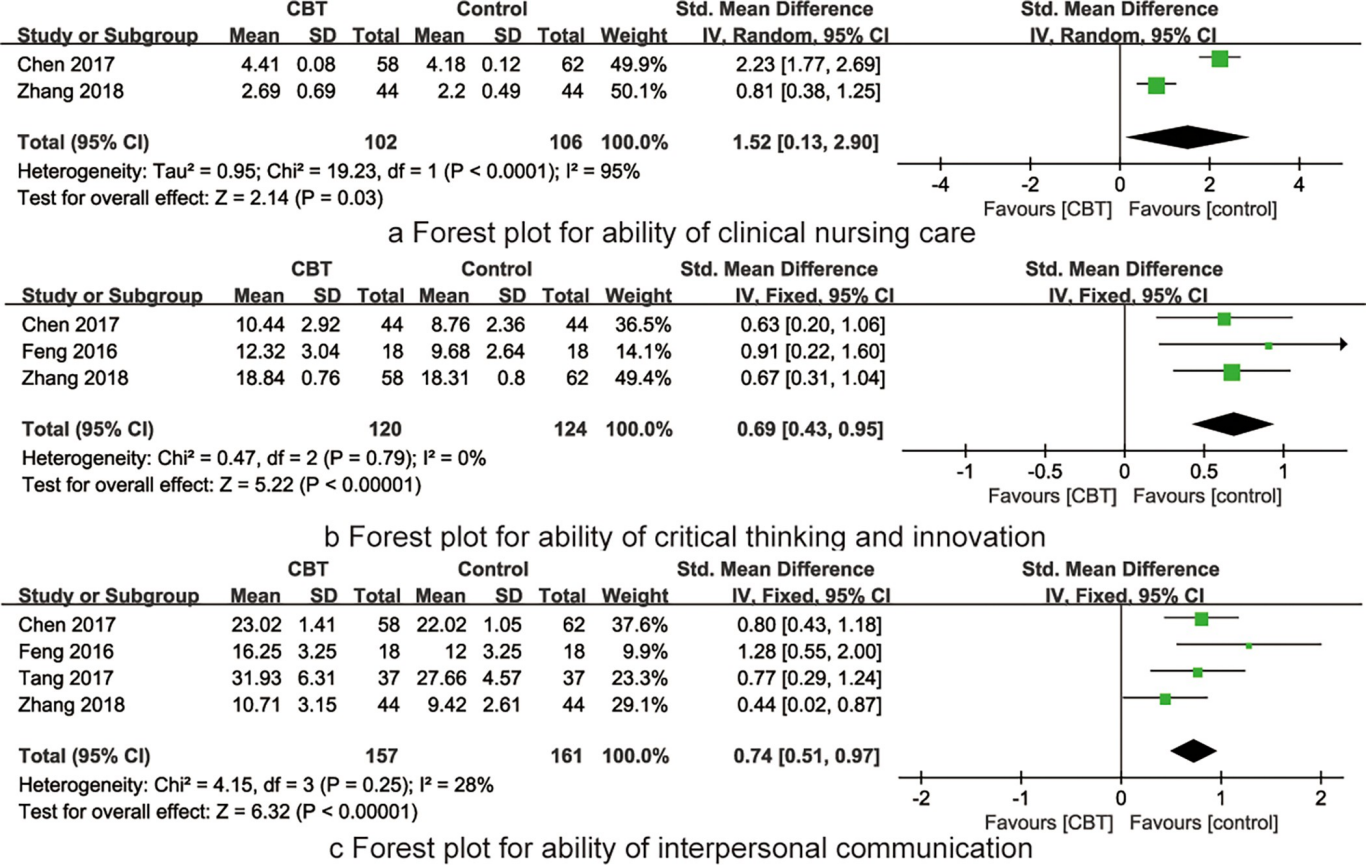
The forest plots of synthesized outcomes.

Two RCTs [17,23] reported the impact of CBT on nurses’ ability of clinical nursing care. The combined results showed high heterogeneity between studies (I^2^ = 95%, P<0.001), so the random effects model was used for meta-analysis. The combined results showed that the ability of clinical nursing care of the CBT group was better than that of the traditional training group, the difference was statistically significant [SMD = 1.52, 95%CI (0.13~2.90), P = 0.03], see Fig 4A.

#### Ability of critical thinking and innovation

Three RCTs [17,20,23] reported the impact of CBT on nurses’ ability of critical thinking and innovation. The combined results showed no heterogeneity between studies (I^2^ = 0%, P = 0.79), so the fixed effects model was used for meta-analysis. The combined results showed that the ability of critical thinking and innovation of the CBT group was better than that of the traditional training group, the difference was statistically significant [SMD = 0.69, 95%CI (0.43~0.95), P<0.001], see Fig 4B.

#### Ability of interpersonal communication

Four RCTs [17,18,20,23] reported the impact of CBT on nurses’ ability of interpersonal communication. The combined results showed low heterogeneity between studies (I^2^ = 28%, P<0.001), so the fix effects model was used for meta-analysis. The combined results showed that the ability of interpersonal communication of the CBT group was better than that of the traditional training group, the difference was statistically significant [SMD = 0.74, 95%CI (0.51~0.97), P<0.001], see Fig 4C.

#### Ability of professional construction and development

**Fig 5:**
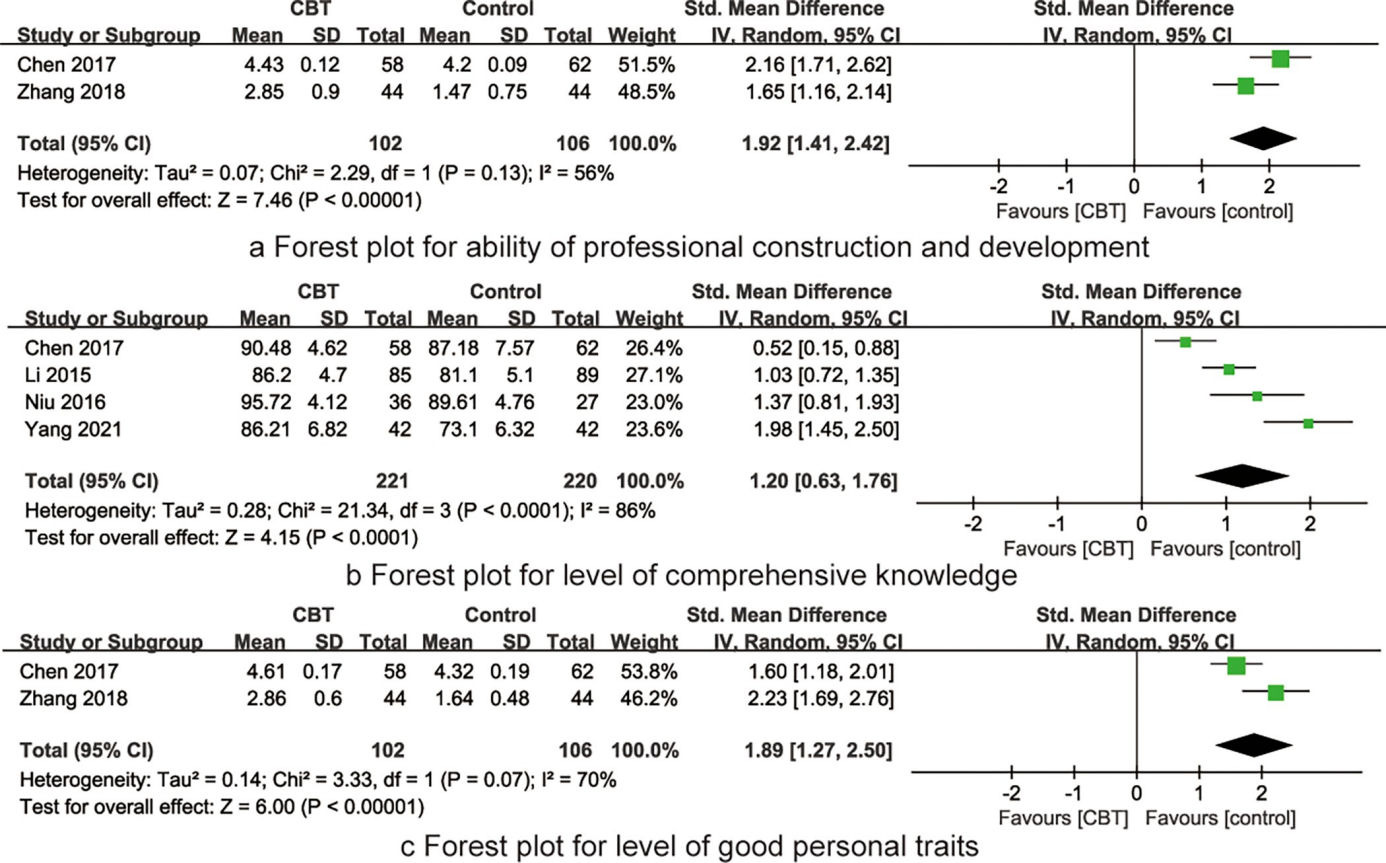
The forest plots of synthesized outcomes.

Two RCTs [17,23] reported the impact of CBT on nurses’ ability of professional construction and development. The combined results showed high heterogeneity between studies (I^2^ = 56%, P = 0.13), so the random effects model was used for meta-analysis. The combined results showed that the ability of professional construction and development of the CBT group was better than that of the traditional training group, the difference was statistically significant [SMD = 1.92, 95%CI (1.41~2.42), P<0.001], see Fig 5A.

#### Level of comprehensive knowledge

Four RCTs [19,21–23] reported the impact of CBT on nurses’ level of comprehensive knowledge. The combined results showed high heterogeneity between studies (I^2^ = 86%, P<0.001), so the random effects model was used for meta-analysis. The combined results showed that the level of comprehensive knowledge of the CBT group was better than that of the traditional training group, the difference was statistically significant [SMD = 1.20, 95%CI (0.63~1.76), P<0.001], see Fig 5B.

#### Level of good personal traits

Two RCTs [17,23] reported the impact of CBT on nurses’ good personal traits. The combined results showed high heterogeneity between studies (I^2^ = 70%, P = 0.07), so the random effects model was used for meta-analysis. The combined results showed that the level of good personal traits of the CBT group was better than that of the traditional training group, the difference was statistically significant [SMD = 1.89, 95%CI (1.27~2.50), P<0.001]], see Fig 5C.

### Sensitivity analysis and publication bias

**Fig 6:**
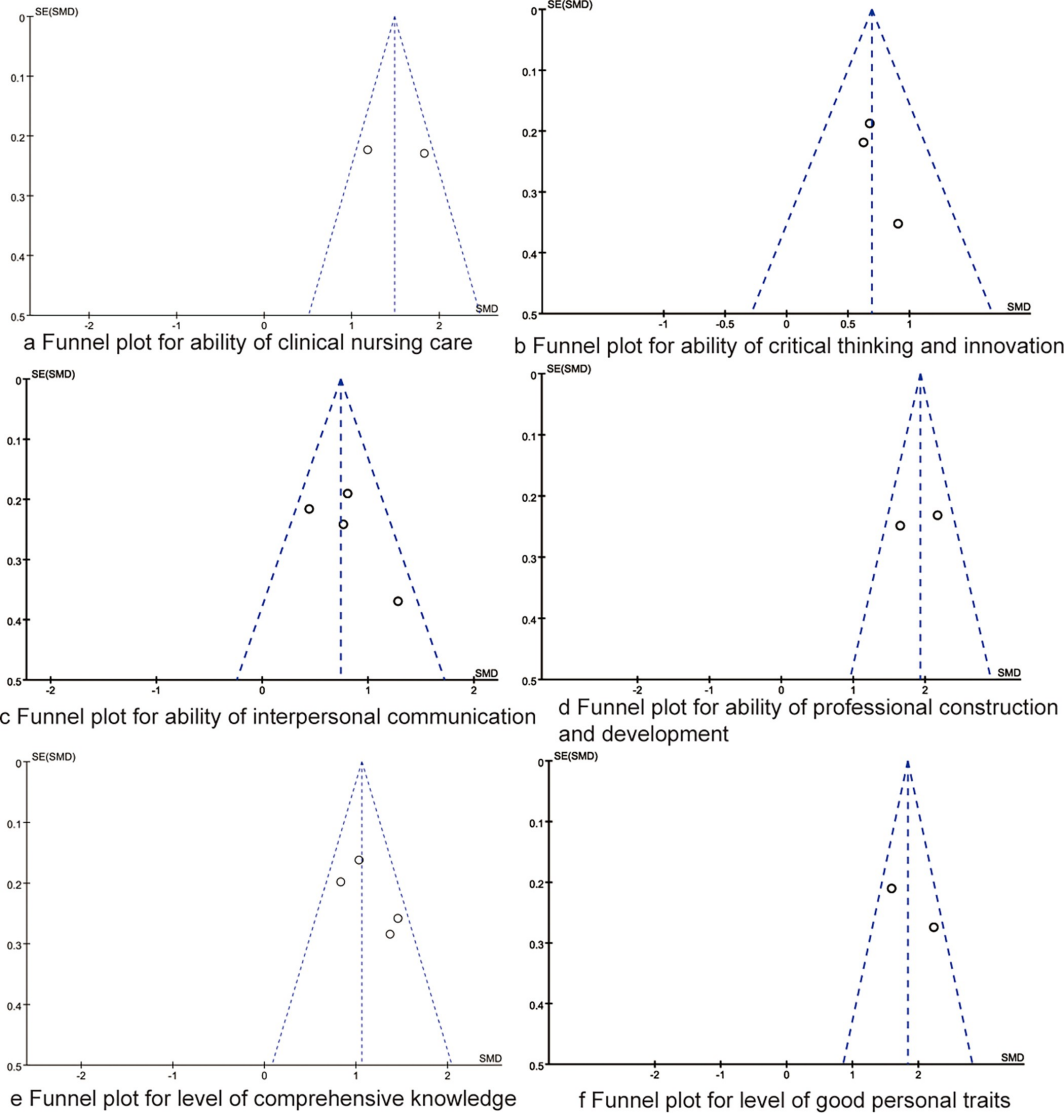
The funnel plots of synthesized outcomes.

We removed individual studies one by one and performed sensitivity analysis. The results showed that the results of the meta-analysis of this study were stable. As shown in Fig 6, the distributions of points in the funnel chart were relatively uniform, the results of Egger regression test indicated that there were no statistical biases amongst the synthesized outcomes (all P>0.05).

## Discussions

Job competency refers to the sum of the ability, skills, knowledge and characteristics of a particular organization that encourages employees to give full play to their potential so as to be competent for the job [24]. Newly recruited nurses are an indispensable part of the clinical nurses. Newly recruited nurses have little nursing experience and safety awareness, less in-depth assessment of the condition, incomplete health education, lack of experience, etc., which can easily lead to adverse nursing events [25,26]. Studies [27,28] have confirmed that the competency of new nurses is a necessary ability for nurses. It is a key link to ensure the quality of nursing service. It can enhance the safety risk awareness of new nurses, improve the predictability of disease changes and the ability to judge the condition, thus being effective to reduce the incidence of adverse events and improve the safety of clinical patients, pre-job training is of great significance for standardizing the nursing skills of newly-practising nurses, improving the competency of nurses, and ensuring the quality and safety of nursing services [29,30]. However, due to the slow development of pre-job training for newly recruited nurses in China, in the system of pre-job training for nurses in China, the training effect is mostly evaluated in a summative manner [31]. The training effect of the newly recruited nurses by the trainer has passed the theoretical examination results and practical operation assessment at the end of training. The main disadvantage is that it ignores the learning situation of nurses during the entire training process and pays more attention to the evaluation of learning results [32]. This has led to the lack of learning initiative of most nurses and dispensable cognition of the training process. At the end and before the exam, a surprise and intensive review can still achieve good results, which is not conducive to the cultivation of the competence and learning ability of the nurses, making the new nursing staff feel uncomfortable when starting the clinical nursing care [33,34]. The results of this present meta-analysis have showed that the CBT is beneficial for improving the ability of the ability of clinical nursing care, critical thinking and innovation, interpersonal communication, professional construction and development, and level of comprehensive knowledge and level of good personal traits. Given the very small sample size and that some meta-analyses were conducted with just two studies, it is hard to make claims that CBT is indeed effective for training new nurses, more work on the effects of CBT needs to be done in the future.

The fundamental task of a competent nurse is to provide effective nursing care to patients. Therefore, nurses must have strong professional knowledge and good operating skills. Besides, their sense of responsibility and clinical nursing ability are also particularly important [35]. The traditional training mode is relatively single, and only stays at the level of experience-based teaching. It usually emphasizes the transfer of knowledge and skills, and the training is not oriented on job competence, which leads to the problem of disconnection between theoretical knowledge and clinical practice for nurses [36]. CBT is to cultivate the key competence of nursing staff. Through tailor-made training plans, the competence level of employees can be improved in a targeted manner, and nurses’ awareness of active learning can be stimulated, and it has a direct effect on behavior, thereby improving the comprehensive knowledge and clinical nursing ability [37–39].

The results of this meta-analysis show that the CBT can effectively improve the good personal characteristics and communication skills of new nurses. Good personal traits refer to the love of nursing profession, the high sense of responsibility and the spirit of being cautious, the spirit of unity and cooperation in work, etc [40]. Interpersonal communication skills refer to the use of good communication skills in nursing practice to provide patients with health knowledge, health care skills, and corresponding psychosocial support [41]. Traditional new nurse training focuses on professional knowledge and skills training, and does not pay too much attention to such issues for new nurses [42]. The CBT training mode based on job competence has gradually shifted from the traditional imparting of knowledge to changing attitudes and values, focusing on personal motivation, work attitude, prudent spirit, communication skills and other competency training practices, which can help them to grow up smoothly and promote the healthy development of new nurses’ professional careers [43,44].

Critical thinking ability and innovation ability, professional construction and development ability are the deeper and more core parts of job competencies. They are important factors that determine nurses’ behavior. To some extent, they will directly affect the quality and development of hospital nursing work [45]. The results of this study show that CBT can improve the critical thinking and innovation abilities, professional construction and development abilities of new nurses. In China, the Confucian educational thought passed down in history and the modern education system that imitated the former Soviet Union’s classroom style have severely restricted the development of students’ creative thinking styles and their professional construction and development capabilities [46,47]. The CBT extends and expands the training content of theory and practice, which is closer to clinical reality, and can effectively improve the ability of new nurses to solve difficult problems and make clinical judgments and decisions, thereby improving their critical thinking skills and the cultivation of innovation ability, professional construction and development ability [48–50].

This meta-analysis has certain limitations that must be considered. Firstly, this study only retrieved Chinese and English literature, and there might be incomplete literature collection. Secondly, the included RCTs were all from China, and there might be certain regional and cultural biases. Different nurses in different places and populations may have different culture background and knowledge, the effects of CBT model may be also different, which need further investigations in various areas and populations. Thirdly, there were some differences in the evaluation indicators of clinical nursing ability, interpersonal communication ability, critical thinking and innovation ability in various studies. Besides, the sample size of included RCTs was small. the small sample size of seven precludes the researchers from making generalizations. Therefore, the results of this study may have certain clinical heterogeneity. Relying solely on heterogeneity alone is not an adequate argument for the use of random effects or fixed effects models, we have both performed analysis for every synthesized outcome by both random effects and fixed effects models, and the results do not substantially change. In the future, large samples and high-quality RCTs are needed to further assess the application effect of CBT.

## Conclusions

In summary, the results of this present meta-analysis have showed that CBT may effectively improve the competency of new nurses, help new nurses not only master knowledge and skills, but also the learning, behavior and abilities. Educational managers may use this training model to train new nurses to improve their job competencies, to be better qualified for nursing jobs, to meet patient care needsmand to ensure the quality of clinical care. However, with only seven studies, it is difficult to acknowledge that the results helped to reconcile the findings in existing literature, more studies on the effects of CBT in clinical nurse training are needed in the future.

## Supporting information

S1 Checklist PRISMA 2020 checklist. (PDF)

S1 File (PDF)

## Abbreviations

CBT: competency-based training
PRISMA: preferred reporting items for systematic reviews and meta-analyses
