# Supplementary material for: The effects of competency-based training model in the training of new nurses: A meta-analysis and systematic review
Source: PLoS One. 2022 Nov 28;17(11):e0277484. doi: 10.1371/journal.pone.0277484 (PMC9704662; doi:10.1371/journal.pone.0277484)
Supplement: S1 File — (PDF) [file pone.0277484.s002.pdf]

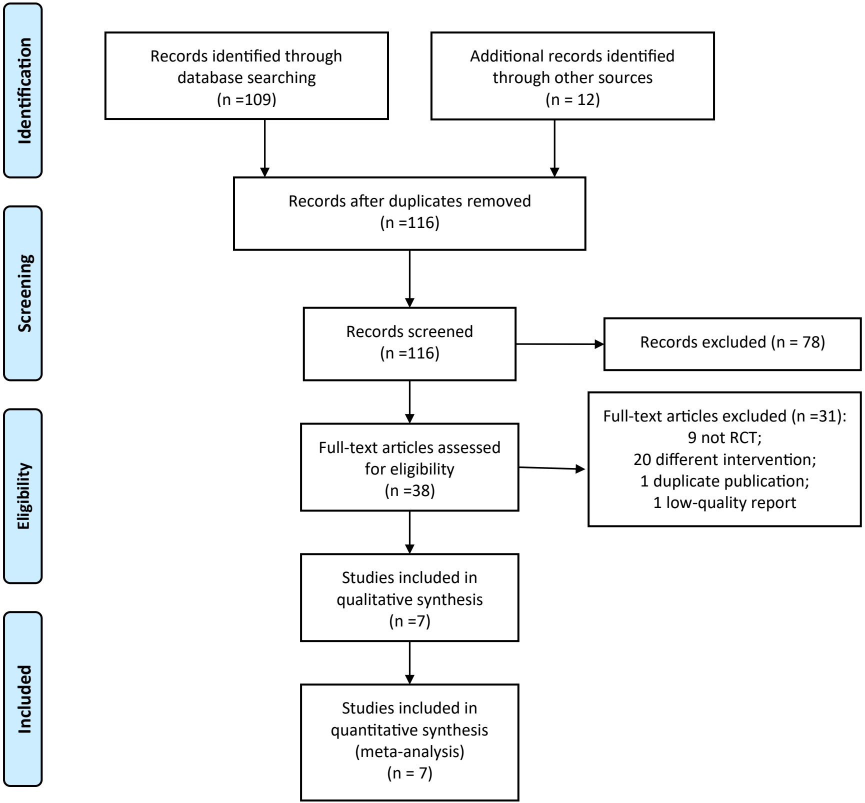
Identification
Screening
Eligibility
Included
Records identified through database searching (n =109)
Additional records identified through other sources (n = 12)
Records after duplicates removed (n =116)
Records screened (n =116)
Records excluded (n = 78)
Full-text articles assessed for eligibility (n =38)
Full-text articles excluded (n =31): 9 not RCT; 20 different intervention; 1 duplicate publication; 1 low-quality report
Studies included in qualitative synthesis (n =7)
Studies included in quantitative synthesis (meta-analysis) (n = 7)
